# Leadless pacemaker implantation in elderly patients with abandoned transvenous pacemakers with depleted batteries

**DOI:** 10.1002/joa3.12866

**Published:** 2023-05-16

**Authors:** William Frick, Yongzhen Chen, Lazarus Zamora, Osama Osman, Ahmed Hussein

**Affiliations:** ^1^ St. Louis University Hospital St. Louis Missouri USA

**Keywords:** elderly, leadless pacemaker, sinus node dysfunction, transvenous pacemakers

## Abstract

This spotlight article gives two clinical case examples for the implementation of a suggested safe and feasible strategy to implant leadless pacemakers instead of changing the generators of transvenous pacemakers with depleted batteries in elderly patients.

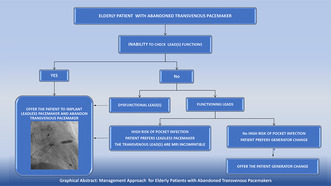

Approximately 80% of transvenous pacemakers are implanted in elderly patients with a reported rate of complications of about 7.4% that include infection, lead dislodgment, pneumothorax, and pocket hematoma. As they are not associated with transvenous leads or surgical pockets, leadless pacemakers have been suggested as a better alternative and have been reported as being safe in very elderly patients aged 85 years or more.^1^

We report two cases of elderly women with abandoned pacemakers with depleted batteries in whom decisions were made to implant leadless pacemakers instead of performing generator change procedure for the existing pacemakers to avoid potential complications.

The first case is a 96‐year‐old female with a history of hypertension, diabetes, dementia, and sinus node dysfunction for which a Boston Scientific INGENIO™ dual chamber pacemaker was implanted in 2001, with the last generator change in 2014 and last documented device interrogation in 2016 quoting battery life of 2.5 years, was admitted to the hospital after an unwitnessed fall in the bathroom. CT head demonstrated a subdural hemorrhage with a 3 mm midline shift for which she did not require surgical intervention per neurosurgery team evaluation. She was found to be confused with episodes of significant sinus bradycardia during which her rate dropped around the low 40s bpm while awake. Her pacemaker could not be interrogated due to battery depletion; therefore, pacemaker leads parameters could not be checked as well. As the fall was suspected to be related to episodes of bradycardia following pacemaker failure and the inability to check the integrity of the pacemaker leads that were more than 20 years old, we offered the patient's family the option to implant a Micra leadless pacemaker instead of attempting generator change. They agreed to the plan, and the implantation procedure was successful with no complications, resulting with the patient becoming less confused. Figure 1 demonstrates the final device position in the presence of abandoned transvenous pacemaker leads.

**FIGURE 1:**
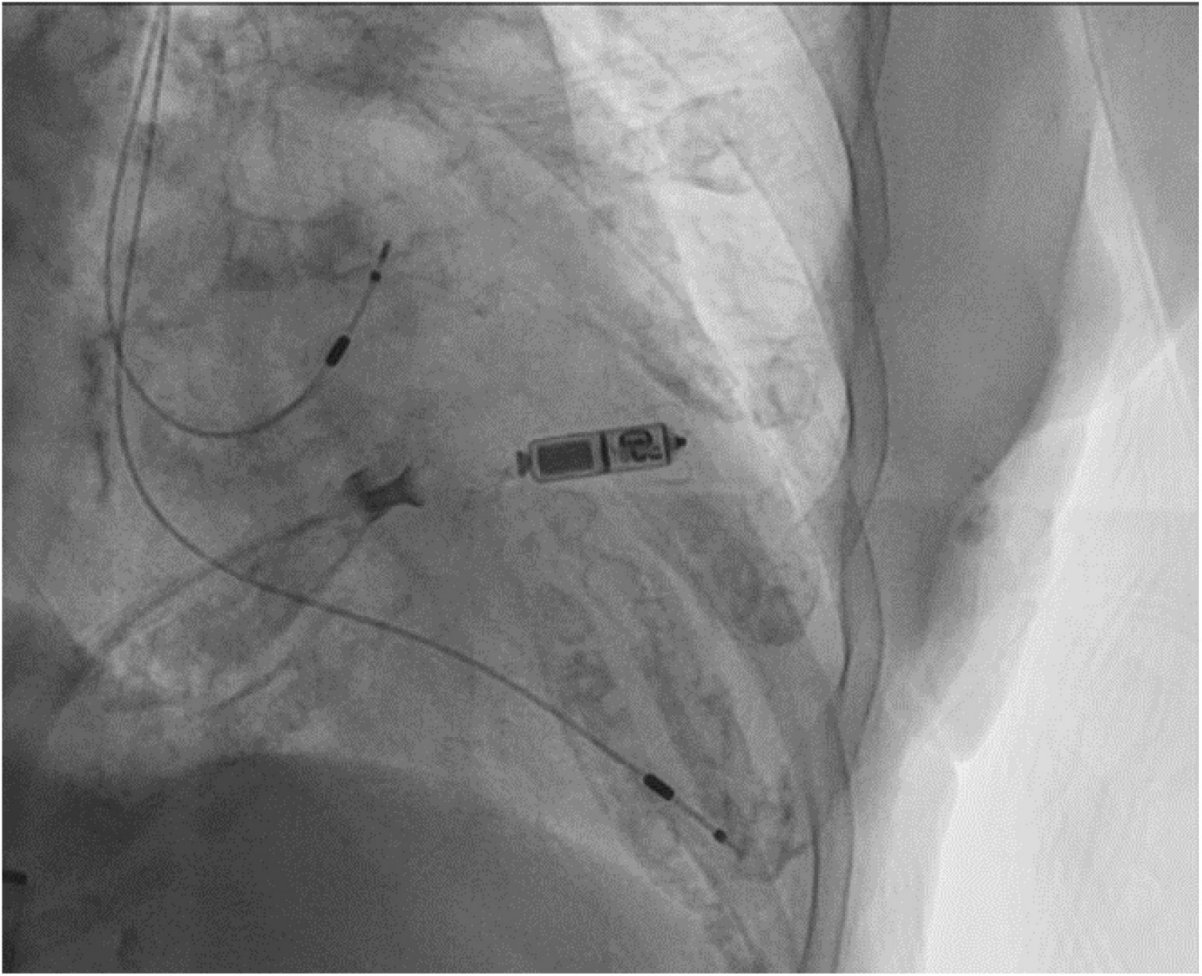
Micra leadless pacemaker implantation in the presence of abandoned transvenous pacemaker leads, right anterior oblique.

The second case is an 81‐year‐old female with a history of Parkinson's disease, hypothyroidism, and single‐chamber Biotronik pacemaker with one right ventricular lead was implanted about 30 years ago for symptomatic sinus node dysfunction with the last generator change reported in 1997 with no available records for subsequent pacemaker interrogation, who presented to the hospital after a fall that was likely caused by a syncopal episode that resulted in a basilar skull fracture with associated subdural and subarachnoid hematomas.

At the time of presentation, she was alert with intermittent confusion. Her electrocardiogram showed sinus rhythm in the 60s, while telemetry review showed prolonged sinus pauses up to 9 s with absent pacing. Similar to the previous case, the pacemaker and lead parameters could not be checked due to battery depletion.

As the syncope was believed to be related to a prolonged sinus pause with the absence of pacing following pacemaker failure, and due to the inability to check the integrity of the pacemaker lead that was approximately more than 30 years old, we discussed with the patient and her family the option of implanting a leadless pacemaker instead of attempting generator change and they agreed to that plan.

The procedure was carried out successfully without complications with subsequent resolution of the patient confusion. Figure 2 demonstrates the final device position in the presence of abandoned right ventricular transvenous pacemaker lead.

**FIGURE 2:**
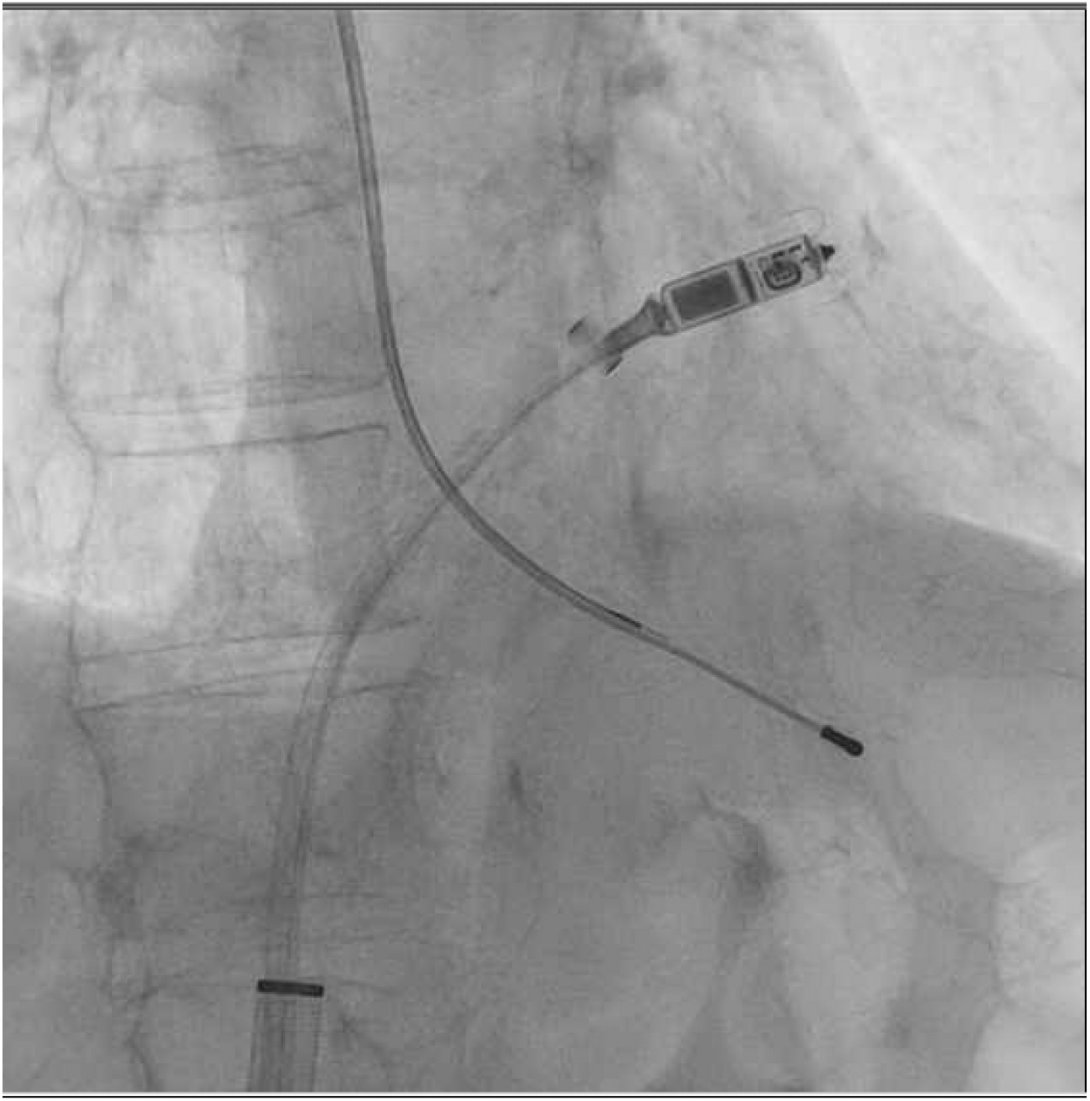
Micra leadless pacemaker implantation in the presence of abandoned transvenous pacemaker lead, right anterior oblique.

In both cases, the Micra leadless pacemaker was implanted in a higher septal position away from the existing right ventricular leads, and interrogation postimplantation demonstrated acceptable sensing, impedance, and pacing threshold with no noise detected. However, it should be noted that higher septal/right ventricular outflow position has thinner myocardium and therefore possibly has higher risk of perforation as it has been previously reported.^2^

Pacemaker battery depletion usually occurs due to loss of pacemaker interrogation follow‐up and leads to reported adverse clinical events in elderly patients.^3^ During the early stage of battery depletion, which is called elective replacement indication and usually lasts for up to 6 months, automatic pacemaker reprogramming occurs and may result in pacemaker syndrome.^4^, ^5^ Following that interval, complete loss of pacemaker functions ensues and the device becomes at the end of service (EOS), and that is usually associated with recurrence of symptoms related to the underlying bradycardia and/or pauses. Pacemakers cannot be interrogated during the EOS status and hence the integrity of their leads, that are more prone to disintegration because of their advanced age, cannot be checked. As such, carrying out pacemaker generator change without checking the leads will carry the risk of connecting the new generator to dysfunctional leads with the persistence of pacing failure. That scenario entails making a surgical wound in a patient with impaired cognition and the need to add one or more leads while anticipating venous access difficulties related to the presence of long‐standing transvenous leads potentially leading to upper extremity central veins stenosis or obstruction.

Therefore, as reported in the two cases presented here, implanting a leadless pacemaker, and leaving the battery depleted pacemaker in situ may be a better alternative approach compared to generator change in elderly patients who are usually at higher risk for pocket infection.^6^

In our patients, the primary motive for abandonment of the old pacemaker in situ and implanting a leadless pacemaker instead of performing generator change was the unknown lead(s) function. However, the utility of this strategy can be extended to elderly patients with functional leads who prefer to avoid the risks associated with generator change as described by Ogano et al.^7^ Other considerations for adopting this strategy include the presence of patient‐related risk factors for pocket infection, such as low body mass index (BMI), corticosteroid use, diabetes, and dementia.^6^ Of note is that our first patient had diabetes and dementia, while the second patient had a low BMI. Also, in the presence of an old magnetic resonance imaging (MRI) incompatible device, as was most likely the case for our two patients, the opportunity to replace that with the Micra leadless pacemaker, which is MRI conditional, needs to be considered to allow for possible future need for MRI studies.

In addition, the strategy we are proposing has proven to be better than a conservative management strategy that avoids invasive interventions altogether, which may be advocated as a result of patients' advanced age, as the leadless pacemaker implantation not only resolves the obvious symptoms related to bradycardia and pauses such as syncope and falls but also the less obvious ones such as confusion, as we previously reported.^8^

From these two cases, we can conclude that leadless pacemaker implantation is a better, feasible, and safe alternative to generator change in elderly patients with abandoned transvenous pacemakers with depleted batteries.

## CONFLICT OF INTEREST STATEMENT

The authors confirm that they have no conflicts of interest in relation to this article.

## ETHICS APPROVAL

N/A.

## PATIENT CONSENT

N/A.

## CLINICAL TRIAL REGISTRATION

N/A.
